# Conducting Randomized Controlled Trials of Complex Interventions in Prisons: A Sisyphean Task?

**DOI:** 10.3389/fpsyt.2022.839958

**Published:** 2022-05-03

**Authors:** Charlotte Lennox, Sarah Leonard, Jane Senior, Caroline Hendricks, Sarah Rybczynska-Bunt, Cath Quinn, Richard Byng, Jenny Shaw

**Affiliations:** ^1^Division of Psychology and Mental Health, Faculty of Biology, Medicine and Health, School of Health Sciences, University of Manchester, Manchester, United Kingdom; ^2^Community and Primary Care Research Group, University of Plymouth, Plymouth, United Kingdom

**Keywords:** prison, randomized controlled trials, interventions, offending, mental health

## Abstract

Randomized Controlled Trials (RCT) are the “gold standard” for measuring the effectiveness of an intervention. However, they have their limitations and are especially complex in prison settings. Several systematic reviews have highlighted some of the issues, including, institutional constraints e.g., “lock-downs,” follow-ups, contamination of allocation conditions and a reliance on self-report measures. In this article, we reflect on our experiences and will describe two RCTs. People in prison are a significantly disadvantaged and vulnerable group, ensuring equitable and effective interventions is key to reducing inequality and promoting positive outcomes. We ask are RCTs of complex interventions in prisons a sisyphean task? We certainly don't think so, but we propose that current accepted practice and research designs may be limiting our understanding and ability to test complex interventions in the real-world context of prisons. RCTs will always have their place, but designs need to be flexible and adaptive, with the development of other rigorous methods for evaluating impact of interventions e.g., non-randomized studies, including pre-post implementation studies. With robust research we can deliver quality evidence-based healthcare in prisons – after all the degree of civilization in a society is revealed by entering its prisons.

## Introduction

Randomized Controlled Trials (RCT) have long been heralded as the “gold standard” for measuring the effectiveness of an intervention, due to their ability to reduce bias and show cause-effect relationships. In this article we will briefly summarize the evidence base for the effectiveness of complex mental health interventions in prison settings, while also identifying the recurrent issues. We will then focus predominantly on our experience of conducting prison-based RCTs and ask the question are prison RCTs of complex interventions a sisyphean task?

To date, there has been a surprising number of systematic reviews of interventions or prisoners/forensic populations (1–21). These reviews have assessed the evidence base in a number of different ways, for example discrete sub-populations [e.g., adolescent offenders (1, 8) female offenders (2, 6, 12)]; offense types [e.g., violent offenses (4, 19)]; specific interventions [e.g., psychotherapy (3, 9, 11)]; or the impact on specific outcomes [e.g., health outcomes, violent behavior or reoffending (10, 12, 14, 21)], with many having a broad inclusion of primary studies designs (9, 12, 13).

Of relevance here are two reviews (17, 21). The first reviewed RCTs of a range of psychological therapies for prisoners with mental health problems (17). Across 37 identified studies, they found a medium effect size for psychological therapies (0.50, 95% confidence interval [0.34, 0.66]), however effects did not appear to be sustained over time. Where trials had used a fidelity measure these were associated with lower effect sizes. The authors also undertook a qualitative analysis of the difficulties of conducting RCTs in prisons. The issues included:

- Post-treatment follow-up - high rates of release, rapid turnover of prisoners, and short duration of stay leading to difficulties with initial recruitment and loss to follow-up.
- Institutional constraints - constraints on the scheduling of sessions, “lock-downs,” high attrition rates partly due to scheduling changes and inmate infractions.
- Small sample sizes.
- Contamination of treatment and control conditions due to the closed communal setting of the prison.
- Not being able to blind the participants to intervention/treatment as usual; and
- Reliance on self-report measures.

The second review examined RCTs of psychological interventions, delivered during incarceration but focused solely on recidivism as the outcome (21). Of 29 RCTs, psychological interventions were associated with reduced reoffending (OR 0.72, 95% CI 0.56–0.92), but after excluding smaller studies there was no significant reduction in recidivism (OR 0.87, 95% CI 0.68–1.11). The number of studies was not large, which the authors suggested supports the evidence that there are significant challenges of doing high-quality research in prisons. Also, many of the studies had a risk of bias, mainly around randomization, intervention deviations and difficulties associated with masking staff and participants to the assigned intervention.

In this context we will now reflect on our own experiences of conducting two prison-based RCTs: Critical Time Intervention (22, 23) and Engager (24, 25). Both studies started with a pilot trial followed by a full RCT. Both interventions were through-the-gate interventions, with baseline assessments completed in prison and then follow-up after release from prison. The two studies are described below and in Table 1.

**Table 1:** Study information for CTI and Engager.

	**CTI**	**Engager**
Date	2007 (pilot trial) 2012–15 (full trial)	2014–15 (pilot trial) 2016–19 (main trial)
Geographical Location	8 prisons – North West England and South East England	3 prisons – North West England and South West England
Inclusion/Exclusion Criteria	Inclusion: • Men (sentenced or remand) • With severe mental illness • In contact with the prison in-reach team • Released from prison within the lifetime of the study • Release would be to an agreed geographical area local to the prison • Severe mental illness was defined as major depressive disorder, hypomania, bipolar disorder and/or any form of psychosis including schizophrenia, schizoaffective disorder and any other non-affective non-organic psychosis. Exclusion: • Did not have severe mental illness • Were to be released outside the agreed geographical discharge area • Posed security/safety issues that would compromise researcher/practitioner safety in prison or the community • Were unable to give informed consent • Had previously participated in the trial during an earlier period in custody.	Inclusion: • Men serving a prison sentence of 2 years or less • With between 4 and 20 weeks remaining until release • Released to the geographical area of the study • Willing to engage with services and research procedures • Were identified as likely to have depression, anxiety, or ptsd currently or following release Exclusion: Men awaiting trial (remand) With severe mental disorder and/or on the caseload of the prison in-reach team Who were under the offender personality disorder pathway service; With active suicidal intent; Who presented a serious risk of harm to the researchers or intervention practitioners Unable to provide informed consent.
Sample Randomized	150	280
Data Collection Points	Baseline (prison) Post-release follow-up – 6 weeks, 6 and 12 months	Baseline (prison) Post-release follow-up – 1, 3, 6, and 12 months
Age; Mean (SD)	36.3 (9.8)	34.5 (10.6)
Ethnicity, *n* (%)^a^ White Ethnic Minority	72 (48) 78 (52)	261 (93) 16 (6)
Most Common Diagnosis (*n*; %)^b^	Schizophrenia (108; 72)	Depression (206; 74)

a *The difference in ethnic minority groups within the two studies reflects the different prisons. Much of CTI recruitment came from the four south east prisons which have higher rates of prisoners from ethnicity minority groups than prisons in the north west and/or south west*.

b *In both studies diagnosis was researcher assessed. In CTI assessed using OPCRIT (Operational Criteria Checklist for Psychotic and Affective Illness) and Engager participants were screened in using the Patient Health Questionnaire-9 (PHQ-9), the Generalized Anxiety Disorder-7 (GAD-7) and the Primary Care PTSD Screen (PC-PTSD)*.

## Critical Time Intervention (CTI)

CTI is an intensive form of mental health case management, operational at times of transition between prison and community and designed for people with severe and enduring mental illness. CTI case managers, routinely mental health nurse, psychologists, or social workers, provided direct care where and when needed, for a limited time period. They began their involvement when the individual was still in prison. For sentenced prisoners, this started 4 weeks before release. For remand prisoners, or those with unpredictable dates of release, intervention starts as soon as the person is known to the prison mental health team. The holistic intervention involves working with the individual and their families (where possible), as well as active liaison and joint working with relevant prison and community services. Five key areas are prioritized: (1) psychiatric treatment and medication management, (2) money management, (3) substance abuse treatment, (4) housing crisis management and (5) life-skills training. CTI is not prescriptive, it responds to the needs of each individual, thus looks slightly different for each person, but still within the five-priority area framework. The intervention includes four phases. Phase 1 is conducted while the person is in prison and requires the development of a tailor-made discharge package based on a comprehensive assessment of the individual's needs. Phases 2 and 3 focus on intensive support post-release and then handing over primary responsibility to community services and phase 4 fully transitioned care to community services to provide long-term support. The aim is that phases 2–4 are completed within 6 weeks of release from prison.

We conducted a multicentre, parallel-group randomized controlled trial across eight English prisons (originally planned for three sites, but additional sites had to be added, discussed below), with follow-up at 6 weeks and 6 and 12 months post-release. A sample of 150 male prisoners were included with eligibility criteria of being: convicted or remanded; cared for by prison mental health teams; diagnosed with severe mental illness, and; with a discharge date within 6 months of the point of recruitment. Of these 150, 72 were randomized to the intervention and 78 were randomized to the usual release planning provided by the prison. Engagement with community mental health teams at 6 weeks was 53% for the intervention group compared with 27% for the control group [95% confidence interval (CI) 0.13% to 0.78%; *p* = 0.012]. At 6 months' follow-up, intervention participants showed continued engagement with teams compared with control participants (95% CI 0.12% to 0.89%; *p* = 0.029); there were no significant differences at 12 months (23).

## Engager

The Engager intervention is designed to engage individuals with common mental health problems in the development of a pathway of care for release and resettlement in the community. It is a manualised, person-centered intervention aiming to address mental health needs as well as to support wider issues including accommodation, education, social relationships, and money management. The intervention is delivered in prison between four- and 16-weeks pre-release and for up to 20 weeks post-release. Experienced support workers and a supervisor with experience of psychological therapy deliver Engager. The practitioner and participant develop a shared understanding of the participant's needs and goals, recognizing the links between emotion, thinking, behavior and social outcomes. A plan is developed, based on agreed goals, and including liaison with relevant agencies and the participant's social networks. A mentalisation-informed approach underpins all elements of the intervention. Use of existing practitioner skills is also key to intervention delivery.

We conducted a two-group parallel randomized superiority trial in three prisons. Men serving a prison sentence of 2 years or less were individually allocated 1:1 to either intervention (Engager plus usual care) or the control (usual care alone) group. The primary outcome was the Clinical Outcomes in Routine Evaluation Outcome Measure (CORE-OM) (26), six months after release. A total of 280 men were randomized (25).

## Our Perspective – What Works?

Intervention allocation in CTI and Engager was at the individual level and so our perspective here focuses on this type of design. However, there are several alternative designs such as cluster, preference and benchmarking controlled trials [we refer the reader to (27, 28)]. Overall, we agree with the reviews (17, 21) in that prison RCTs are possible. In both studies participant engagement was positive, with high levels of consent and enthusiasm for the interventions, but also being involved in the research process. However, the unique prison context can make standard trial procedures and standard assessments of study quality more difficult to achieve.

### Pilot Trials

In both our studies we undertook pilot trials. For CTI (22) the focus of the pilot was very much about testing if the intervention could produce an outcome, while in Engager (24) the pilot trial explicitly examined trial design and recruitment building on earlier feasibility work (29), but importantly also had an embedded realist^1^-informed formative process evaluation, which focused on how the intervention was working (30). Both pilot trials provided invaluable knowledge and supported the development of relationships with the recruitment sites. On reflection, had the CTI pilot (22) formally tested recruitment and eligibility rates, then perhaps we could have better predicted the slow recruitment rates faced and negated the need to add so many other sites. Slow recruitment was due to a complex interplay of lengthy delays in approval and other operational delays such as change in healthcare providers, which meant that men became ineligible to take part due to not being released within the study period.

The difference between these two pilot studies also reflects the fast pace of change we have seen in our understanding of intervention development and testing, and the improved guidance on feasibility and pilot trials (31). The UK Medical Research Council (MRC) published a framework on developing and evaluating complex interventions in 2000 (32), it was revised in 2006 (33), but has been very recently updated again in 2021 (34) – clear evidence of this fast pace. In addition, our theorical understanding of acceptability, often a key outcome in feasibility and pilot trials, has advanced with the work of Sekhon (35), using this framework may have added significant depth of understanding of the anticipated and experienced acceptability from the perspective of the intervention delivers and recipients.

### Blinding

Single-, double-, and triple-blinding are commonly used in RCTs. A single-blind study blinds the participant from knowing which study trial arm they have been assigned. A double-blind study blinds both the participants and researchers to allocation. And triple-blinding involves blinding the participants, researchers, and statistician.

The review above (17) highlighted that blinding was problematic. Blinding participants where the intervention is a psychological therapy and/or person facing is difficult, if not impossible. In CTI (23), we were able to blind the researcher and statistician data. We were able to blind the researcher to allocation as there was no face-to-face contact with the participants after baseline data collection, which was before participants were randomized. In Engager, we were only able to blind the statistician. In our Engager pilot trial (24) we tested and reported on our attempts to blind the researchers, but researchers were unblinded very quickly. Due to the frequent contact the researchers had with participants, participants were keen to share their experiences with the researchers and/or the researchers saw the participants with the Engager Practitioners due to the closed confines of the prison. We considered a range of workable solutions to maintain blinding, such as using a article-based self-complete outcome measure for participants but decided against this due to literacy problems and the likely increase in incomplete data. In the main Engager trial (25) the researchers knew trial arm allocation, this was a positive in that it allowed for the continued building of rapport between the researcher and participant to facilitate follow-up rates but may have diluted the relationship building effects of the intervention. Both studies could have considered adaptations to their design to allow recruitment to each arm to be staggered, but this lengthens the overall study time and cost.

### Outcome Measures

How we measure outcomes in forensic populations is notoriously complicated and the reason why there is little agreement about which outcomes to use (36). Forensic settings and forensic populations are diverse. For example, settings can include, police custody, prison, probation services in the community and secure forensic hospitals. Even within the same setting there is diversity, for example, secure forensic hospitals have different security levels and different provider organizations. Services may also be viewed as having diverse goals including clinical, legal and public safety. In addition, forensic populations may have multiple and varied problems. For example, personality disorder, mental illness, learning disability, substance abuse and offending behavior, with many co-occurring, leading to many combinations of potentially relevant outcomes.

To confound this further there are also different type of outcomes. Objective outcome measures can be viewed as outcomes such as rehospitalisation, reoffending and death, and are usually obtained from administrative datasets. In our CTI study (23) our primary outcome was based on information collected from participants electronic health records. While on the surface this would seem to avoid the limitations associated with self-report data e.g., social desirability, honesty, introspective ability, latent nature of the measures, missing data, it was not without shortcomings. The data was only as good as the quality of the written records, and at times this was poor, something highlighted by other researchers (37). We also planned to supplement this with information from UK health registries, however due to accessibility issues, likely data quality and an inability to join data from different registries, we were unable to progress this. A recent systematic review of 160 RCTs accessing routinely collected heath data, found only a very small proportion of UK RCTs (about 3%) and highlighted issues with access, quality and a lack of joined-up thinking between the registries and the regulatory authorities (38). In both CTI and Engager we had planned to obtain offending data, but faced similar issues to the health data in terms of protracted approval processes.

Over recent years there has been an explosion of the number of subjective outcomes available. There have been a number of reviews (36, 39, 40) of outcome measures in forensic settings, identifying a large number of questionnaire-based instruments, focusing mainly on risk and clinical symptoms, neglecting quality of life, functional outcomes and patient involvement. In the most recent review, a total of 435 measures were identified. Of the 10 most frequently used, half of the instruments were primarily focused on risk. Only one instrument, the Camberwell Assessment of Need: Forensic Version (CANFOR) (41), had adequate evidence for its development and content validity.

In our Engager trial (25), outcome data was primarily subjective and significant work went into deciding which outcomes to use, with the aim of selecting a set of outcome measures that captured the most important areas of the Engager intervention. We adopted a four-stage approach involving; a single round Delphi survey to identify the most important outcome domains; a focused review of the literature, testing of these measures in the target population to assess acceptability and the psychometric viability of the measures and a consensus panel meeting to select the primary outcome measure for the trial and key secondary outcome measures. In addition, we actively sought the input of our Peer Research Group (42) throughout this process. After the four stages the CORE-OM (26) and CANFOR (41) both received the same number of votes to be the primary outcome measure. We opted for the CORE-OM (26) as the primary outcome measure. It had marginally superior psychometric properties, could be administered in a highly scripted fashion that would reduce researcher bias, some items were of little relevance to a prison population and there were issues with the CANFOR being able to demonstrate change over-time (43, 44). There is also some criticism of the reliability of the scoring system for the CANFOR. We had considered using outcomes based on practitioner records, however, it quickly became clear that these were not recorded in a sufficiently consistent way to merit inclusion. They were not undertaken at set time points, were often subjective in terms of focus, and suffered from missing data.

Ultimately, even going through this process of selecting the primary outcome, we found problems with the CORE-OM. The before and after changes for individuals did not match the journey of rehabilitation and recovery detailed in the depth process evaluation (45), where we found that the intervention was more effective when practitioners developed an in-depth understanding of the participant. It may therefore not to be sensitive enough to detect small unpredictable steps in recovery resultant from the intervention for individuals with lifelong experiences of adversity. It also highlights the problems of reducing very complex interventions down to just one outcome, it may be that we just do not have adequate outcomes to test such complex interventions. We tried to use the PSYCLOPS (46) questionnaire, an idiographic measure designed to detect changes in person specific problems, but the prison environment rendered it unworkable because once released individuals' problems were almost entirely different.

### Intervention Fidelity

One of the reviews highlighted above showed that studies including a measure of fidelity were associated with lower effect sizes (17). Intervention fidelity, like outcomes, is a complex area with a lack of agreement about the appropriate indicators of fidelity and how these should be measured (47, 48). It is argued that any assessment of fidelity should look at the intervention designer-, provider- and recipient-levels (49). However, it is likely that the delivery of an intervention as complex, person-centered and flexible to the individual as CTI or Engager will be harder to evaluate than simpler “one dose fits all” designs.

In CTI, fidelity was assessed using an adapted version of the fidelity scale used in the Critical Time Intervention – Task Shifting study (50) at eight time points over the course of the trial. However, a more reliable and detailed way to assess fidelity would have been for the CTI manager to complete a checklist per participant against the core CTI principles. This would have allowed more detailed analysis of what each participant received, mapped against their needs. There was variation in fidelity to the intervention across the different CTI managers.

In Engager, fidelity was assessed by creating an intervention delivery timeline which depicts practitioner and supervisor start and end dates, instances of training sessions, research team-engager supervisor supervision and periods of prison “lockdown,” where practitioners were unable to access the prison sites to deliver the intervention. Practitioners and supervisors also kept records of contacts in the form of daily activity logs (documenting time spent with participants, or activities related to participants e.g., arranging appointments, liaison with other services) and recorded session case notes (documenting intervention delivered and received).

We recognized however that this only measures superficial aspects of fidelity (reach and dose) and not the multiple mechanisms designed to be at play in such a complex intervention (30). There is, however, little published regarding fidelity in complex behavioral interventions and there needs to be more published on fidelity results (51).

### Process Evaluation

The biggest difference between CTI and Engager was the complexity and depth of the qualitative components. In CTI, we undertook a nested qualitative study. At that time even this was relatively unheard of in RCTs (52). Jump five-years and we were undertaking one of the most in-depth process evaluations for complex health interventions (30, 45). Even after the publication of the MRC guidance in 2000 and 2006, process evaluations have often been small qualitative add-ons to trials and of little importance to the main trial findings, although more recent guidance emphasizes the importance of detailed analysis (53). The parallel mixed method process evaluation in Engager not only provided evidence of breadth and depth, and from multiple perspectives about what was delivered to participants, but also allowed us to focus in on how team dynamics and underlying beliefs and values affected implementation, and to propose what might be done to support practitioners further to optimize delivery. Documenting suboptimal implementation, was important for trial result interpretation and development of future practice. The use of realist-informed methods allowed us to interrogate the intervention mechanisms by assessing if delivering the specified intervention components produce the hypothesized outcomes. This gave us insight into how the intervention can have a sustained effect when delivered well. We showed how consistent delivery across time could lead to the several mechanisms being activated, often repeatedly, to achieve incremental but sustainable change (25, 45). It also allowed us to examine more deeply what “meaningful change” meant for the intervention participants in ways that standard outcome measures cannot assess.

## Discussion

Are conducting RCTs of complex interventions in prisons: A Sisyphean task?

No, far from it. In our experience they can be conducted, are a key tool in developing evidence-informed practice and for some interventions provide the best approach to test effectiveness. But there is also a need for flexibility so that we are not unduly limited by a specific set of perspectives. For us there are some key must dos. Pilot and/or feasibility trials to help minimize risks to the main trial e.g., ensuring testing of recruitment and follow-up rates, developing effective relationships with the prisons so they see the value of research. A robust process evaluation is key, for understanding what was delivered but more importantly how was it delivered and how it produces change, how interventions work has often received little attention in prison research.

Areas where we need to improve are our understanding how best to assess fidelity and our choice of outcome measures, is this user led vs standardized measures vs. bespoke, or should we use a combination. Plus, we need to work to improve access to routinely collected data, other European countries, such as the Nordic countries are much more advanced here. We also need to work with the prison system to ensure they see the value in supporting independent, external research to reduce protracted approvals. We must not get overly fixed on some traditional aspects of rigor. Alongside flexible adaptive RCTs we also propose the development of rigorous methods for evaluating impact of interventions in non-randomized studies e.g., pre-post implementation studies. Before-after health or quality of life questionnaire data can be examined alongside processes of care, economic data and depth qualitative process evaluation analyses. Where novel interventions are adopted as treatment as usual there is a place for robust service evaluations of routinely collected data, where research ethics would not be required.

It was Fyodor Dostoyevsky who said: “The degree of civilization in a society is revealed by entering its prisons” and therefore we continue to undertake prison research, despite some of its challenges. We strive to reduce health inequalities and drive-up quality healthcare for a group of people who are significantly disadvantaged and vulnerable (54–57) so that we can live in a more civilized society.

## Data Availability Statement

The original contributions presented in the study are included in the article, further inquiries can be directed to the corresponding author.

## Author Contributions

CL drafted the first version of the article. SL, JS, CH, SR-B, CQ, RB, and JS revised the article. All authors accepted the final version of the article. All authors contributed to the article and approved the submitted version.

## Funding

RB received support from the National Institute for Health Research Applied Research Collaboration South West Peninsula. CTI was funded by the National Institute for Health Research, Service Delivery and Organisation Programme (09/1004/15). Engager was funded by National Institute for Health Research Programme Grant for Applied Research Programme (RP-PG-1210-12011).

## Conflict of Interest

The authors declare that the research was conducted in the absence of any commercial or financial relationships that could be construed as a potential conflict of interest.

## Publisher's Note

All claims expressed in this article are solely those of the authors and do not necessarily represent those of their affiliated organizations, or those of the publisher, the editors and the reviewers. Any product that may be evaluated in this article, or claim that may be made by its manufacturer, is not guaranteed or endorsed by the publisher.

## Footnotes

^1^Realist evaluations are theory driven and focus on evaluating “what works in which circumstances and for whom?”, rather than merely ‘does it work?.
